# Apoaequorin differentially modulates fear memory in adult and aged rats

**DOI:** 10.1002/brb3.1832

**Published:** 2020-09-18

**Authors:** Vanessa L. Ehlers, Chad W. Smies, James R. Moyer

**Affiliations:** ^1^ Department of Psychology University of Wisconsin‐Milwaukee Milwaukee WI USA; ^2^ Department of Biological Sciences University of Wisconsin‐Milwaukee Milwaukee WI USA

**Keywords:** aging, calcium, calcium‐binding protein, hippocampus, trace fear conditioning

## Abstract

**Introduction:**

Cognitive deficits during aging are pervasive across species and learning paradigms. One of the major mechanisms thought to play a role in age‐related memory decline is dysregulated calcium (Ca^2+^) homeostasis. Aging is associated with impaired function of several calcium‐regulatory mechanisms, including calcium‐binding proteins that normally support intracellular Ca^2+^ regulation. This age‐related calcium‐binding protein dysfunction and changes in expression lead to disrupted maintenance of intracellular Ca^2+^, thus contributing to memory decline. Other work has found that age‐related cognitive deficits can be mitigated by either blocking Ca^2+^ entry into the cytosol or preventing its release from intracellular Ca^2+^ stores. However, the effect of calcium‐binding protein administration on cognitive function during aging is not well‐understood. Our laboratory has previously shown that the calcium‐binding protein apoaequorin (AQ) is neuroprotective during oxygen–glucose deprivation, a model of in vitro ischemia characterized by calcium‐induced excitotoxicity. The current experiments assessed the effect of direct dorsal hippocampal AQ infusion on trace and context fear memory in adult and aged rats.

**Methods:**

Adult (3–6 months) and aged (22–26 months) male F344 rats were randomly assigned to different experimental infusion groups before undergoing trace fear conditioning and testing. In experiment 1, rats received bilateral dorsal hippocampal infusions of either vehicle or AQ (4% w/v) 24 hr before trace fear conditioning. In experiment 2, rats received bilateral dorsal hippocampal infusions of either vehicle or 4% AQ 1 hr before trace fear conditioning and 1 hr before testing.

**Results:**

Aged rats displayed impaired trace and context fear memory. While a single AQ infusion 24 hr before trace fear conditioning was insufficient to rescue age‐related trace fear memory deficits, AQ infusion 1 hr before both conditioning and testing abolished age‐related context fear memory deficits.

**Conclusions:**

These results suggest that intrahippocampal infusion of AQ may reverse aging‐related deficits in hippocampus‐dependent context fear memory.

## Graphical abstract

Two experiments were conducted to evaluate the impact of direct intrahippocampal infusion of the calcium binding protein apoaequorin (AQ) on trace and context fear memory. Experiment 1 involved a single infusion prior to training and experiment 2 involved an infusion prior to training and testing. Trace and context fear memory were impaired in aged rats relative to adult rats. Direct intrahippocampal infusion of AQ prior to both conditioning and testing reversed the aging‐related context fear memory deficits.

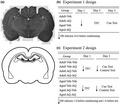

## 1 INTRODUCTION

Aging is accompanied by cognitive decline even in the absence of overt pathology. The study of learning and memory deficits in aged animals can provide a useful laboratory model to understand the neurobiological mechanisms that contribute to this decline. Aging deficits in rodents are largely evident in behavioral tasks that require an intact medial temporal network for processing spatiotemporal and sensory information, and include spatial navigation (Barnes, 1979; Foster, Defazio, & Bizon, 2012; Guidi, Kumar, Rani, & Foster, 2014; Pereira et al., 2014; Tombaugh, Rowe, & Rose, 2005; Yu, Curlik, Oh, Yin, & Disterhoft, 2017), object recognition (Bartolini, Casamenti, & Pepeu, 1996; Burke, Wallace, Nematollahi, Uprety, & Barnes, 2010; de Lima et al., 2005; Pieta Dias et al., 2007; Pitsikas, Rigamonti, Cella, Sakellaridis, & Muller, 2005; Vannucchi, Scali, Kopf, Pepeu, & Casamenti, 1997), eyeblink conditioning (Deyo, Straube, & Disterhoft, 1989; Graves & Solomon, 1985; Kishimoto, Suzuki, Kawahara, & Kirino, 2001; Knuttinen, Gamelli, Weiss, Power, & Disterhoft, 2001; Moyer, Power, Thompson, & Disterhoft, 2000; Solomon & Groccia‐Ellison, 1996; Thompson, Moyer, & Disterhoft, 1996), context fear (Houston, Stevenson, McNaughton, & Barnes, 1999; Kaczorowski, Davis, & Moyer, 2012; Kaczorowski & Disterhoft, 2009), and trace fear conditioning (Dulka, Pullins, Cullen, Moyer, & Helmstetter, 2020; McEchron, Cheng, & Gilmartin, 2004; Moyer & Brown, 2006; Villarreal, Dykes, & Barea‐Rodriguez, 2004). These studies strongly implicate aging as a major contributing factor to impaired learning of these behavioral tasks.

The calcium (Ca^2+^) hypothesis of aging proposes that Ca^2+^ dysregulation disrupts neuronal function and signaling processes that are necessary for cognitive operations like learning and memory (Khachaturian, 1987, 1994; Landfield, 1987). Several calcium‐regulatory mechanisms (including ion channels, intracellular Ca^2+^ stores, and calcium‐binding proteins), display altered expression and/or function in the aged brain. In the aged hippocampus, increased L‐type voltage‐dependent Ca^2+^ channel (L‐VDCC) density is negatively correlated with spatial water maze learning (Thibault & Landfield, 1996). Application of ryanodine, which blocks calcium‐induced Ca^2+^ release, impedes glutamate‐induced Ca^2+^ elevation in aged neurons, suggesting age‐related increases of Ca^2+^ transients are due to increased Ca^2+^ release from intracellular stores (Clodfelter, Porter, Landfield, & Thibault, 2002). Importantly, this work was conducted in cultured neurons, thus somewhat limiting translatability to intact aging animals. Nevertheless, it highlights the influence that intracellular Ca^2+^ stores might have on dysregulated Ca^2+^ during aging. These mechanisms also interact; calcium‐induced Ca^2+^ release can influence L‐VDCC activity, while Ca^2+^ influx via L‐VDCCs leads to calcium‐induced Ca^2+^ release (Chavis, Fagni, Lansman, & Bockaert, 1996).

Calcium‐binding proteins, which show reduced expression during aging, also play a fundamental role in maintaining neuronal Ca^2+^ homeostasis by buffering intracellular Ca^2+^. Calbindin‐D28k is an EF‐hand calcium‐binding protein that is reduced in aged rat and rabbit dentate gyrus (de Jong et al., 1996) and in middle‐aged and aged rat perirhinal cortex (Moyer, Furtak, McGann, & Brown, 2011). Calretinin, another EF‐hand calcium‐binding protein, is reduced in aged human cortex (Bu, Sathyendra, Nagykery, & Geula, 2003). Functionally, calcium‐binding proteins are implicated in several physiological and molecular signaling processes that are crucial for learning. Hippocampal synaptic plasticity is impaired in the absence of calretinin (Schurmans et al., 1997) and calbindin (Klapstein et al., 1998; Molinari et al., 1996). Application of brain‐derived neurotrophic factor (BDNF) increases calbindin expression in cortical cultures (Fiumelli, Kiraly, Ambrus, Magistretti, & Martin, 2000; Widmer & Hefti, 1994) and hippocampal cultures (Ip, Li, Yancopoulos, & Lindsay, 1993), suggesting endogenous calcium‐binding proteins participate in neuronal growth and differentiation. Furthermore, downregulated calcium‐binding protein expression during aging is thought to increase vulnerability to calcium‐mediated cytotoxicity (Iacopino & Christakos, 1990), and to contribute to the pathogenesis and neuronal degeneration present in Alzheimer's disease (for review, see Fairless, Williams, & Diem, 2019; Kook et al., 2014; Riascos et al., 2011). Reduced expression of these and other calcium‐binding proteins in old age may therefore leave cells more susceptible to Ca^2+^ dysregulation and thus may contribute to overt cognitive deficits.

Several studies suggest a link between reduced calcium‐binding protein expression and cognitive and neuronal impairment. Neurons from calbindin‐deficient adult mice exhibit impaired long‐term potentiation (LTP) (Jouvenceau et al., 2002), and active place avoidance learning is impaired in both adult and middle‐aged calbindin‐deficient mice when compared to wild‐type (Moreno et al., 2012). Expression levels are closely tied to behavioral performance: while aged mice with reduced hippocampal calbindin display impaired object recognition memory, aged mice whose calbindin expression is similar to younger adults do not (Soontornniyomkij et al., 2012). Other studies suggest that restoring Ca^2+^ regulation, either by blocking Ca^2+^ influx via L‐VDCCs (Deyo et al., 1989; Veng, Mesches, & Browning, 2003) or Ca^2+^ release from intracellular stores (Gant et al., 2015; Hopp et al., 2014), facilitates learning in aged animals. However, the role of calcium‐binding protein administration in rescuing aging‐related cognitive decline has not been closely examined.

To determine whether calcium‐binding protein administration mitigates age‐related cognitive deficits, we examined the effect of dorsal hippocampal infusion of the calcium‐binding protein apoaequorin (AQ) on trace fear conditioning, a form of associative learning that has previously been used to study age‐related learning impairment (McEchron et al., 2004; Moyer & Brown, 2006; Villarreal et al., 2004). The AQ protein and coelenterazine are components of the photoprotein aequorin, which has a similar protein structure to other EF‐hand calcium‐binding proteins (Inouye et al., 1985), and has traditionally been used as a Ca^2+^ indicator (for review, see Blinks, 1978; Shimomura, Kishi, & Inouye, 1993). Our laboratory has previously demonstrated that AQ is neuroprotective when it is bilaterally infused into the dorsal hippocampus either 24 or 48 hr prior to an in vitro ischemic insult (Detert, Adams, Lescher, Lyons, & Moyer, 2013), which normally produces cell death via calcium‐mediated toxicity (Kristian & Siesjo, 1998). Given that age‐related cognitive decline is associated with Ca^2+^ dysregulation as well as reduced expression of endogenous calcium‐binding proteins, the goal of the present study was to determine whether administration of the AQ protein would mitigate learning deficits in aged animals.

In our first experiment, we investigated whether a single bilateral dorsal hippocampal infusion of AQ given 24 hr prior to a trace fear conditioning session was capable of improving deficits observed in aged rats. In our second experiment, we examined whether bilateral dorsal hippocampal infusions of AQ delivered 1 hr prior to training and/or 1 hr prior to testing was capable of improving or reversing any memory deficits observed in the aged rats. This allowed us to examine both the immediate effects of a single AQ infusion and any potential state‐dependent effects of AQ on adult and aged rats. Our results indicate that while a single intrahippocampal infusion of AQ was unable to ameliorate aging‐related trace fear memory deficits, intrahippocampal AQ infusions given both 1 hr prior to training and 1 hr prior to testing were capable of reversing context fear memory deficits in the aged rats.

## 2 METHODS

### 2.1 Subjects

Adult (3–6 months) and aged (22–26 months) male F344 rats were housed individually with access to food and water ad libitum. Rats were maintained on a 14‐hr light/10‐hr dark cycle (lights on at 7 a.m.) in an Association for Assessment and Accreditation of Laboratory Animal Care (AAALAC) accredited facility. All experimental procedures were carried out in accordance with NIH guidelines and approved by the University of Wisconsin‐Milwaukee animal care and use committee (ACUC).

### 2.2 Surgery

**Figure 1:**
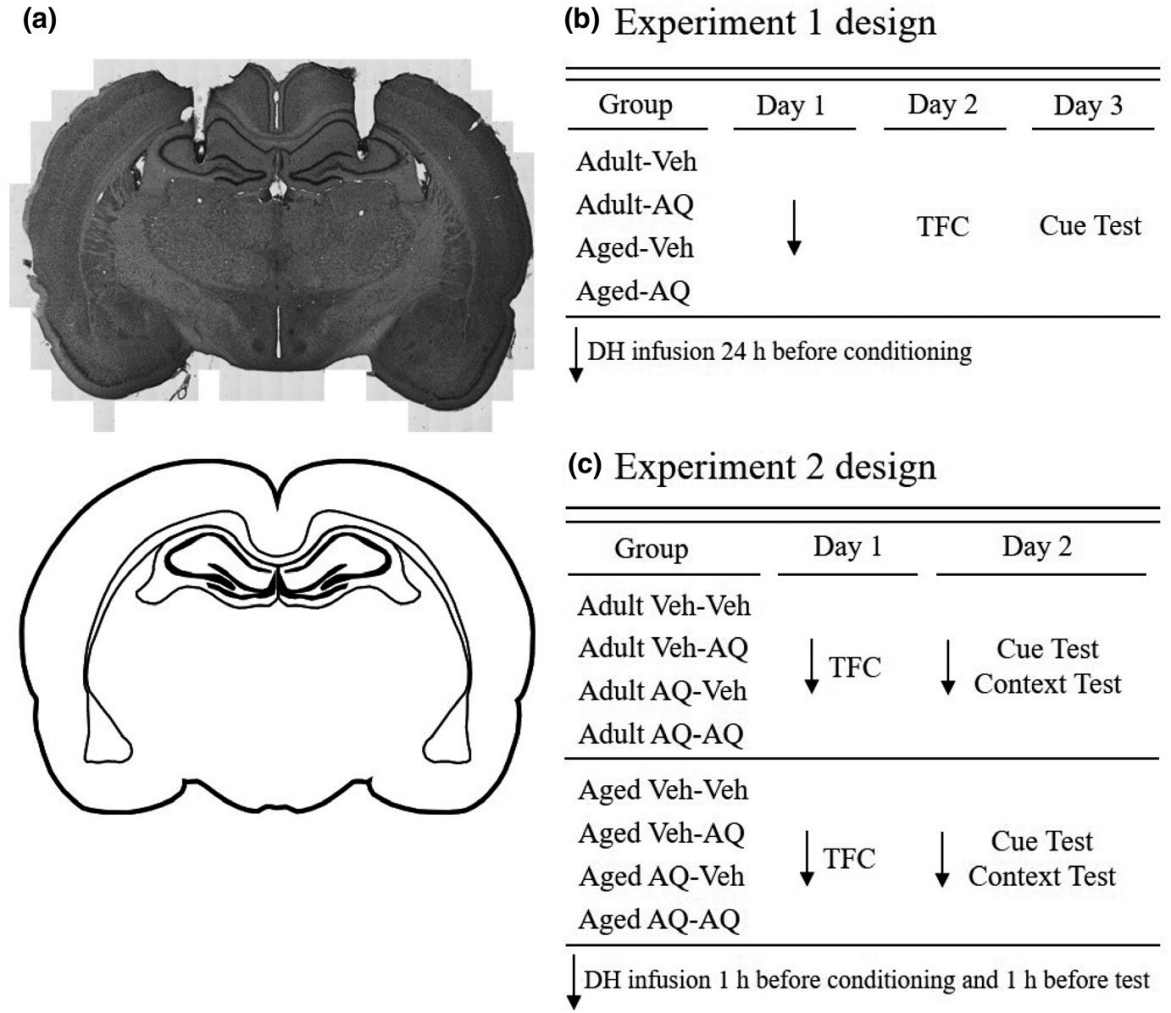
Representative dorsal hippocampal infusions and experimental design. (a) *Top:* Stitched 10x brightfield image of coronal brain section stained with cresyl violet showing representative cannula placements in dorsal hippocampus. *Bottom:* Representative schematic showing approximate location for cannulation surgeries. This schematic corresponds most closely to Paxinos & Watson plate 55, or −2.64 mm relative to Bregma (Paxinos & Watson, 2007). (b) Experimental design for experiment 1. Adult and aged rats were randomly assigned to two different infusion groups: Veh or AQ. On day 1, rats received bilateral infusions directly into dorsal hippocampus. On day 2, rats received a single 10 trial session of auditory trace fear conditioning. On day 3, a cue test was conducted in a novel chamber. (c) Experimental design for experiment 2. Adult and aged rats were randomly assigned to one of four infusion groups: Veh‐Veh rats received vehicle before both training and testing; Veh‐AQ rats received vehicle before training and AQ before testing; AQ‐Veh rats received AQ before training and vehicle before testing; and AQ‐AQ rats received AQ before both training and testing. On day 1 rats received bilateral dorsal hippocampal infusions 1 hr before a single 6 trial trace fear conditioning session. On day 2 rats received bilateral dorsal hippocampal infusions 1 hr before an auditory cue test in a novel context to assess CS and trace fear memory. Thirty minutes following the cue test rats were placed back into the training chamber for 10 min to assess context fear memory. Abbreviations: vehicle (Veh); apoaequorin (AQ)

Rats were mounted on a stereotaxic apparatus under isoflurane‐induced anesthesia. Bilateral stainless steel guide cannula (26‐gauge) were lowered into the dorsal hippocampus using stereotaxic coordinates (AP −3.5 mm, L ± 2.6 mm, V −3.0 mm) relative to Bregma (see Figure 1; Detert et al., 2013). Stainless steel screws and acrylic cement were used to secure the cannula to the skull. Plastic caps were screwed onto the guide cannula to prevent occlusion. Rats were given oral Carprofen (5 mg/kg) for pain management and were allowed a minimum of 7 days of recovery before infusions.

### 2.3 Drugs and Infusions

Apoaequorin (4% w/v; CalciGenix) was infused into the dorsal hippocampus in zero Ca^2+^ artificial cerebral spinal fluid (aCSF; in mM: 124.00 NaCl, 2.80 KCl, 2.00 MgSO_4_, 1.25 NaH_2_PO_4_, 26.00 NaHCO_3_, 10.00 D‐glucose, and 0.40 Na‐ascorbate) with 6% DMSO added to facilitate neuronal uptake. This dose of AQ was chosen based on a previously published study from our laboratory indicating a 4% intrahippocampal infusion of AQ mitigated ischemic cell death in hippocampal neurons (Detert et al., 2013). Control infusions consisted of aCSF and 6% DMSO. Infusions (0.5 µl/hemisphere) occurred over 60 s, and infusion cannula were removed 2 min after infusion to ensure diffusion away from the tip. The 33‐gauge infusion cannula extended 0.5 mm beyond the guide cannula. Infusions were performed in a room that was isolated from both the colony room as well as the behavioral training and testing room. To habituate rats to infusion procedures, they were transported to the infusion room one day before the first infusion, where infusion cannulae were lowered and remained in place for 60 s while running the infusion pump.

### 2.4 Fear conditioning and testing chambers

Plexiglas and stainless steel rectangular chambers (30.5 × 25.4 × 30.5 cm; Coulbourn Instruments) set up within sound‐attenuating boxes were used for trace fear conditioning. A standard grid floor consisting of 26 parallel steel rods (5 mm in diameter, 6 mm spacing) was connected to a precision adjustable shock generator (Coulbourn Instruments) to deliver a scrambled footshock unconditional stimulus (US). A constant background noise of ~58 dB (measured daily by a sound meter, A scale; model: Digital 2055, RadioShack) was provided by a ventilation fan within the sound‐attenuating chamber. A miniature incandescent white lamp (28 V, type 1819, illumination 1.1 lux) was used to illuminate the chamber. Prior to training, the chamber was wiped with a 5% ammonium hydroxide solution. The room lights were left on for the entire training session (illumination 20.9 lux).

The auditory cue test session was conducted in separate Plexiglas chambers, which were located within separate sound‐attenuating boxes in the same room as the training chambers. The test chambers were octagonal with a black‐painted Plexiglas floor and clear Plexiglas walls. The chambers were illuminated using infrared light. To provide a different olfactory stimulus from that used during training, the test chamber walls were wiped with 2% acetic acid, and the tray beneath the floor was filled with clean bedding before each test session. Room lights remained off (illumination 0.2 lux) during the test session. For context fear memory test sessions, the original training chamber served as the context test chamber. Room lights remained on throughout the context test. FreezeFrame 4.01 (Actimetrics Software, Coulbourn Instruments) was used to control stimulus delivery during training and testing.

The activity of each rat during training and testing was recorded by a remote CCD video camera (model #STC‐MB33USB; Sensor Technologies America, Inc. Carrollton, TX) mounted to the top of each behavioral chamber, and the video data were fed to a PC running FreezeFrame 4.01. Data were analyzed using FreezeView 4.01 (Actimetrics Software). Freezing was defined as the absence of all movement except that required for respiration (Blanchard & Blanchard, 1969), and a 1 s bout of immobility was scored as freezing. To measure trace fear acquisition during training we analyzed percent freezing during the trace interval of each trial (i.e., the 30 s period following the conditional stimulus (CS) offset). Cued fear memory was determined by calculating average percent freezing during the CS of both trials and trace interval of both trials, and subtracting baseline freezing (percent freezing during the first two min of the test) from the CS (ΔCS) and trace interval (ΔTI). Context fear memory was assessed by calculating percent freezing during the first five min of the context test.

### 2.5 Procedure

#### 2.5.1 Experiment 1: AQ infusion 24 hr before trace fear conditioning

We first examined the effect of a single dorsal hippocampal AQ infusion on trace fear memory (experimental design is shown in Figure 1b). Adult and aged rats were randomly assigned to receive bilateral infusions of either vehicle (Adult‐Veh, *n* = 5; Aged‐Veh, *n* = 7) or AQ (Adult‐AQ, *n* = 5; Aged‐AQ, *n* = 6) 24 hr before trace fear conditioning. Handling occurred for at least 1 week prior to the conditioning session. On day 1, rats received bilateral infusions directly into the dorsal hippocampus. On day 2, rats were transported to the behavior room and placed in the training chambers. Training consisted of a single 10‐trial session of auditory trace fear conditioning, using a 15 s 80 dB white noise as the CS, followed by a 30 s trace interval, and a 1 s, 1 mA scrambled footshock US. Each trial was separated by a 5.2 min (±20%) intertrial interval (ITI) to maximize CS and minimize context conditioning (Detert, Kampa, & Moyer, 2008). On day 3, 24 hr after conditioning, rats were placed in the novel context to test auditory cued fear memory. Immediately following a 2‐min baseline, fear memory was assessed by calculating the average percentage of time spent freezing (percent freezing) during two CS‐alone trials separated by a 5.2 min (±20%) ITI.

#### 2.5.2 Experiment 2: AQ infusion 1 hr before trace fear conditioning and/or 1 hr before test

In our second experiment (design shown in Figure 1c), we examined the effects of AQ infusion 1 hr before training, 1 hr before testing, or both 1 hr before training and 1 hr before testing. We randomly assigned adult and aged rats to one of four experimental groups: Veh‐Veh (infusion of vehicle before training and testing, adult *n* = 9, aged *n* = 9); Veh‐AQ (pretraining vehicle infusion, and pretesting AQ infusion, adult *n* = 5, aged *n* = 4); AQ‐Veh (pretraining AQ infusion, and pretesting vehicle infusion, adult *n* = 5, aged *n* = 4); and AQ‐AQ (infusion of AQ before training and testing, adult *n* = 5, aged *n* = 4).

Similar to our first experiment, handling was conducted for at least 1 week prior to training. On day 1, bilateral dorsal hippocampal infusions occurred 1 hr before trace fear conditioning. This conditioning paradigm was identical to that of the previous experiment (15 s 80 dB white noise CS, 30 s trace interval, 1 s 1 mA scrambled footshock US, 5.2 min (±20%) ITI) except the number of trials was reduced from 10 to 6, because pilot data from an initial cohort demonstrated extremely robust baseline freezing during the auditory cue test session (data not shown). On day 2, rats received bilateral infusions 1 hr before behavioral testing began. Rats were first tested for auditory cued fear memory in a novel context. After a 2 min baseline, rats were given two CS‐alone presentations (15 s 80 dB white noise, 30 s trace interval, 2.9 min ITI). Average percent freezing during the trace interval of those two trials was used to assess trace fear memory. Thirty minutes after the cue test, rats received a 10‐min context test in the original training context. No CS or US stimuli were presented during this test. A pilot experiment was conducted to determine whether test order affected behavioral outcome for either age‐group, in the absence of AQ infusion. Based on this, we determined aging‐related context fear memory deficits were greatest when rats underwent a cue test in a novel context before undergoing a context test in the original training context. Since the goal of the present study was to determine whether AQ infusion would mitigate any aging‐related deficits of fear memory, we chose to conduct the cue test first to maximize the occurrence of aging‐related context fear memory deficits. Thus, any observed behavioral modifications are likely the result of the AQ protein, as opposed to confounding effects of test order. Following testing, rats were returned to their home cages.

### 2.6 Baseline activity measures

To evaluate any age‐ or infusion‐related differences in gross baseline activity, the number of grid crossings was determined during the first minute of the trace fear conditioning session (prior to any CS or US presentations) for each rat. A grid of two evenly spaced lines was overlaid on the computer monitor while playing back the video during this first minute of the training session. A grid crossing was defined as movement of the anterior third of the rat (i.e., forepaws) across any grid line. Total number of grid crossings and latency to freezing onset were recorded by an experimenter who was blind to age and infusion group.

### 2.7 Analysis

Overall treatment effects were examined using Student's *t* test, repeated‐measures ANOVA or two‐factor ANOVA where appropriate using SPSS 23.0 (IBM Corp.). A Greenhouse–Geisser correction was used if Mauchly's test of Sphericity indicated the assumption of sphericity had been violated. Fisher's protected least significant difference (PLSD) was used for post hoc analysis when main effects were significant (*α* = 0.05), and for multiple pairwise comparisons. Data are expressed as mean ± 1 *SEM*.

## 3 RESULTS

### 3.1 Experiment 1: AQ infusion 24 hr before trace fear conditioning

**Figure 2:**
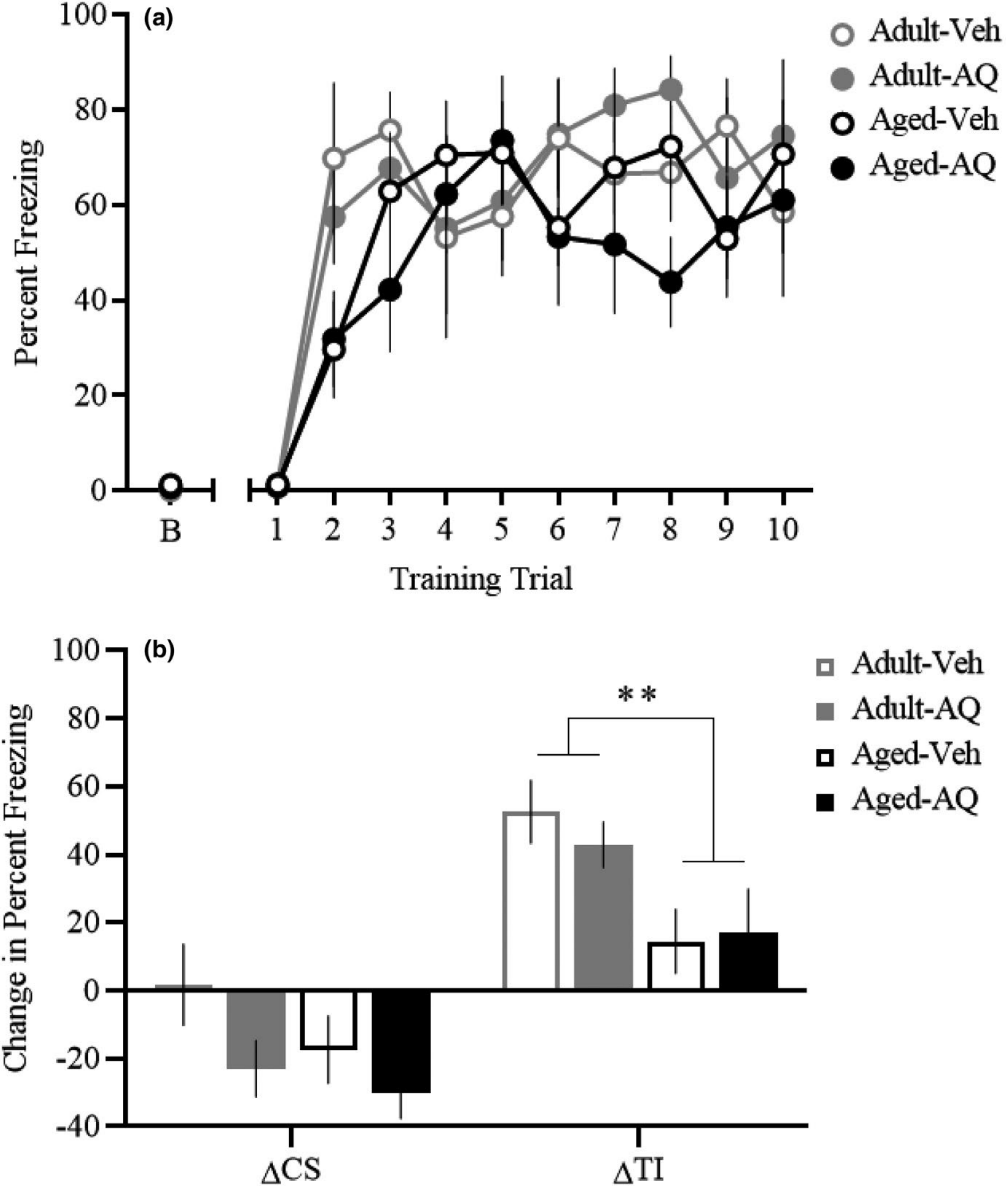
Apoaequorin (AQ) infusion 24 hr before trace fear conditioning does not affect trace fear memory. (a) Rats received a single 10 trial session of auditory trace fear conditioning 24 hr after receiving dorsal hippocampal infusions of either vehicle or AQ. Trace fear acquisition was similar between groups. (b) Twenty‐four hours after trace fear conditioning, rats underwent a cue test in a novel context. There was a significant main effect of age on ΔTI freezing, such that aged rats froze less than adults. There was no effect of infusion on ΔTI freezing. ΔCS freezing was unaffected by either age or infusion. Abbreviations: percent freezing during baseline (b); percent freezing during the conditional stimulus minus baseline (ΔCS); percent freezing during the trace interval minus baseline (ΔTI); **p < .01

In experiment 1, we examined whether a single dorsal hippocampal infusion of AQ administered 24 hr prior to trace fear conditioning would improve fear memory deficits in aged rats. During the conditioning session, behavioral performance was similar between groups (Figure 2a). Repeated‐measures ANOVA of percent freezing during the trace interval revealed a significant effect of trial [*F*(9, 171) = 16.0, *p* < .001], an interaction between age and trial [*F*(9, 171) = 2.21, *p* < .05], but neither an infusion by trial interaction [*F*(9, 171) = 0.25, *p* = .99], nor an age by infusion by trial interaction [*F*(9, 171) = 0.91, *p* = .52] were observed. Despite the interaction effect between trial and age, there was no main effect of age [*F*(1, 19) = 1.78, *p* = .2], no effect of infusion [*F*(1, 19) = 0.15, *p* = .7], and no age by infusion interaction [*F*(1, 19) = 0.49, *p* = .49]. During the auditory cue test the following day, paired *t* tests within each group revealed that Adult‐Veh and Adult‐AQ rats display significantly greater freezing during the trace interval (Adult‐Veh: 79.3 ± 13.5; Adult‐AQ: 75.0 ± 12.8) relative to baseline (Adult‐Veh: 26.7 ± 10.2; Adult‐AQ: 32.0 ± 11.5; both *p*‐values, *p* < .01), while aged rats did not exhibit a significant increase in trace interval freezing (Aged‐Veh: 45.6 ± 13.1; Aged‐AQ: 53.7 ± 13.5) relative to baseline (Aged‐Veh: 31.0 ± 7.5, *p* = .17; Aged‐AQ: 36.7 ± 8.3, *p* = .25), suggesting that trace fear retrieval is intact in adult rats, but significantly impaired in aged rats. Behavioral performance during the cue test was assessed by calculating the difference between baseline freezing and average freezing during the CS (ΔCS) and trace interval (ΔTI) to accommodate any individual differences in baseline freezing (Dulka et al., 2020). Analysis of ΔCS freezing revealed no significant effects of age [*F*(1,19) = 1.79, *p* = .2] or infusion [*F*(1,19) = 3.66, *p* = .07], and a nonsignificant interaction [*F*(1,19) = 0.38, *p* = .55]. In contrast, analysis of ΔTI freezing revealed a significant overall aging deficit [*F*(1,19) = 9.62, *p* < .01] (see Figure 2b). There was no effect of infusion [*F*(1,19) = 0.12, *p* = .73] nor was there an age by infusion interaction effect [*F*(1,19) = 0.35, *p* = .56] on ΔTI freezing. Together, these data suggest that when a single intrahippocampal infusion of AQ is given 24 hr before conditioning, it does not affect trace fear memory in the adult rats, nor does it eliminate the trace fear memory deficit observed in the aged rats.

To account for any confounds due to aging‐related changes in hyperactivity, which have been reported in some studies (see Deyo et al., 1989), grid crossings were analyzed during the first minute of the training session, prior to any CS or US presentations (see Table 1). No effects of age [*F*(1, 19) = 1.41, *p* = .25] or infusion [*F*(1, 19) = 3.17, *p* = .09] were observed, nor was there an age by infusion interaction [*F*(1, 19) = 0.52, *p* = .48]. Similarly, when latency to onset of freezing during the cue test was analyzed, there was no effect of age [*F*(1, 19) = 1.91, *p* = .18], infusion [*F*(1, 19) = 0.14, *p* = .72], or an age by infusion interaction [*F*(1, 19) = 0.02, *p* = .89]. These data suggest that the trace fear memory deficit observed in the aged rats did not merely result from an aging‐related increase in hyperactivity.

**Table 1:** Grid crossings and latency to onset of freezing for experiment 1

Group	No. of grid crossings	Latency to onset of freezing (s)
Adult‐Veh	13.4 ± 0.4	14.2 ± 4.3
Adult‐AQ	16.3 ± 2.0	16.7 ± 2.9
Aged‐Veh	11.8 ± 1.6	21.8 ± 6.4
Aged‐AQ	12.5 ± 0.8	23.0 ± 3.8

Note

Values are means ± *SEM*. To evaluate whether baseline activity levels differed due to either infusion or age, grid crossings were analyzed during the first minute of the trace fear conditioning session. Latency to onset of freezing during the cue test was used to determine whether there was any effect of either age or infusion on freezing commencement. There was no effect of age or infusion on either measure.

### 3.2 Experiment 2: AQ infusion 1 hr prior to trace fear conditioning and 1 hr prior to testing

**Figure 3:**
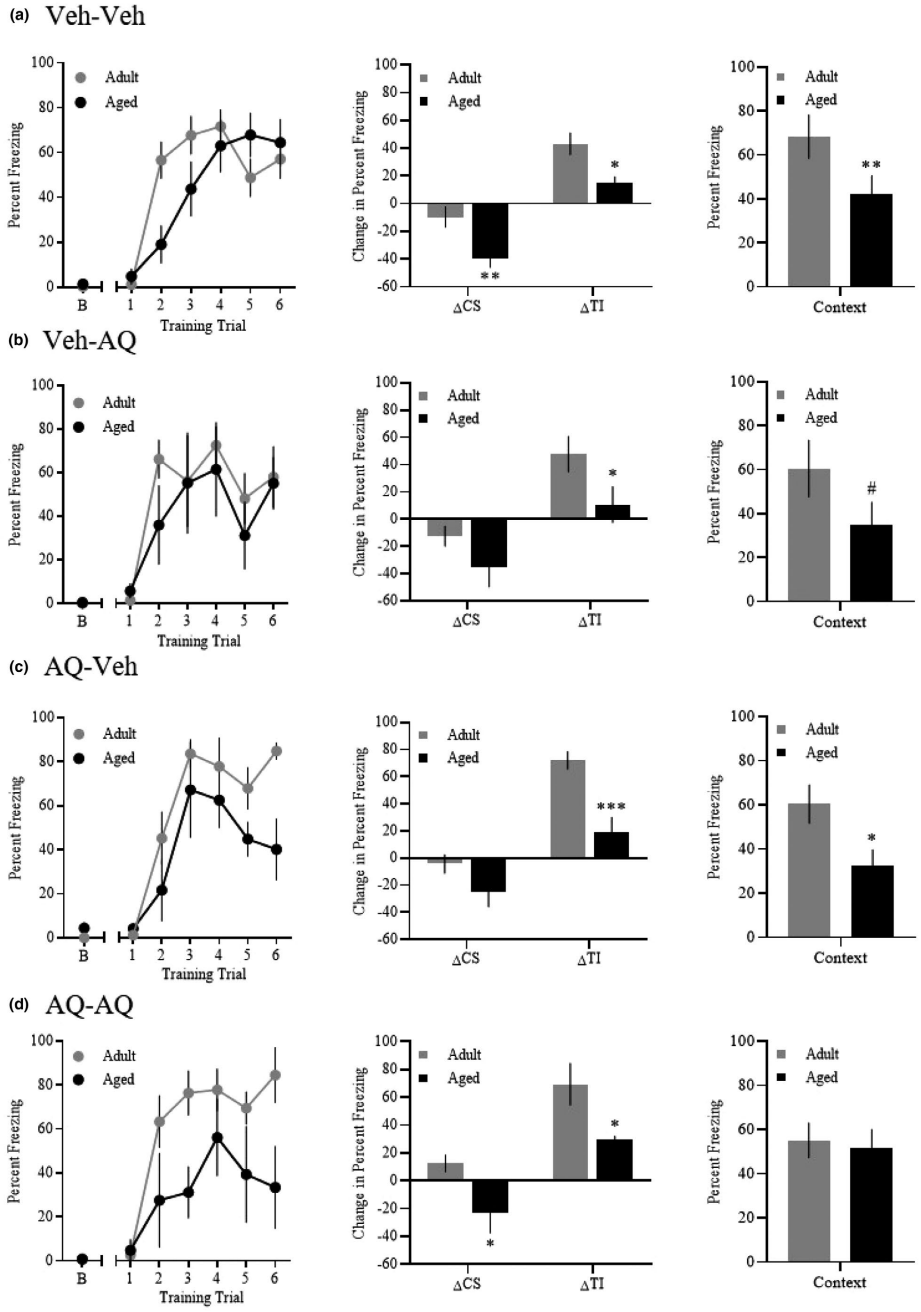
Apoaequorin (AQ) infusion 1 hr before trace fear conditioning and 1 hr before testing rescues an age‐related context fear memory deficit. (a) Training (left graph) and test (right two graphs) data for rats infused with vehicle only (Veh‐Veh). Aged rats demonstrate impaired ΔCS and ΔTI freezing during the cue test, and impaired freezing during the context test. (b) Training (left graph) and test (right two graphs) data for rats infused with vehicle before training and AQ before testing (Veh‐AQ). Aged rats display impaired ΔTI freezing during the cue test, and a trending impairment in freezing during the context test. (c) Training (left graph) and test (right two graphs) data for rats infused with AQ before training and vehicle before testing (AQ‐Veh). Aged rats display impaired ΔTI freezing during the cue test, as well as impaired freezing during the context test. (d) Training (left graph) and test (right two graphs) data for rats infused with AQ only (AQ‐AQ). Aged rats display impaired ΔCS and ΔTI freezing during the cue test, but freezing during the context test is similar to that of adults (*p* = .796). Abbreviations: percent freezing during baseline (b); percent freezing during the conditional stimulus minus baseline (ΔCS); percent freezing during the trace interval minus baseline (ΔTI); #*p* = .06; *p < .05; **p < .01; ***p < .001

In experiment 2, we investigated whether dorsal hippocampal AQ infusion 1 hr before trace fear conditioning and/or 1 hr before testing rescued aging‐related fear memory deficits. During the conditioning session on day 1 (see Figure 3), rats displayed increased freezing across training trial [*F*(3.44, 127.18) = 37.25, *p* < .001], but there was no significant interaction between trial and age [*F*(3.44, 127.18) = 2.42, *p* = .06], no interaction between trial and infusion [*F*(10.31, 127.18) = 0.73, *p* = .7], and no trial by age by infusion interaction [*F*(10.31, 127.18) = 1.08, *p* = .39].

To determine whether adult rats within each infusion group demonstrate intact trace fear memory, we performed paired *t* tests comparing average baseline freezing to average trace interval freezing during the cue test within each infusion group, separated by age. We determined that all adult infusion groups demonstrated a significant increase of trace interval freezing (Veh‐Veh: 79.5 ± 7.6; Veh‐AQ: 75.4 ± 18.0; AQ‐Veh: 82.4 ± 6.7; AQ‐AQ: 72.1 ± 15.8) relative to baseline (Veh‐Veh: 36.4 ± 9.4, *p* < .001; Veh‐AQ: 27.6 ± 9.2, *p* < .05; AQ‐Veh: 10.3 ± 5.6, *p* < .001; AQ‐AQ: 2.7 ± 1.6, *p* < .01). Similar pairwise *t* tests among aged rats interestingly revealed a significant increase of trace interval freezing relative to baseline for Veh‐Veh and AQ‐AQ rats, but not for Veh‐AQ or AQ‐Veh rats (Veh‐Veh TI: 65.7 ± 5.7, B: 50.6 ± 6.8, *p* < .01; Veh‐AQ TI: 53.7 ± 18.3, B: 43.0 ± 14.5, *p* = .48; AQ‐Veh TI: 48.6 ± 5.7, B: 29.8 ± 14.4, *p* = .2; AQ‐AQ TI: 60.0 ± 10.8, B: 29.8 ± 12.7, *p* < .001). This could suggest that aged rats display state‐dependent enhancement of trace fear memory (i.e., only when brain states at the time of encoding and retrieval are similar); however, this analysis does not indicate whether aged rats perform similarly to their adult counterparts, and thus, whether aging‐related trace fear memory deficits are actually abolished.

Several effects became apparent during the cue and context tests on day 2 (Figure 3).

Similar to experiment 1, behavioral performance during the cue test was assessed by calculating the difference between baseline freezing and average freezing during the CS (ΔCS) and TI (ΔTI). First, a two‐way ANOVA revealed ΔCS freezing was significantly reduced for aged rats relative to adults [*F*(1, 37) = 17.18, *p* < .01]. Pairwise comparisons were performed to examine specifically which infusion groups displayed an aging deficit, and revealed aged Veh‐Veh and aged AQ‐AQ rats displayed significantly reduced ΔCS freezing relative to their adult counterparts (Veh‐Veh, *p* < .01; AQ‐AQ, *p* < .05). Second, ΔTI freezing was significantly reduced for aged rats relative to adults [*F*(1, 37) = 32.3, *p* < .001]. Pairwise comparisons revealed that aged rats in each infusion group displayed reduced ΔTI freezing when compared to their respective adult controls (Veh‐Veh, Veh‐AQ, and AQ‐AQ *p* < .05; AQ‐Veh, *p* < .001). These data suggest that aged rats display impaired trace freezing during the cue test and that AQ infusion 1 hr before either acquisition or testing or both does not rescue this deficit.

Analysis of average percent freezing over the entire 10‐min context test revealed a significant main effect of age [*F*(1, 37) = 27.32, *p* < .001], but no main effect of infusion [*F*(3, 37) = 0.8, *p* = .5], and no age by infusion interaction [*F*(3, 37) = 0.92, *p* = .44]. Notably, freezing within the control group (adult Veh‐Veh) peaked within the first 5 min of the context test and began to decrease thereafter, suggesting extinction learning begins at this timepoint. Therefore, we also analyzed age and infusion effects within these first 5 min. Analysis of average percent freezing during the first 5 min of the context test revealed a significant effect of age [*F*(1, 37) = 11.08, *p* < .01]. In order to better understand how age affected performance during the context test, we performed pairwise comparisons within each infusion group. We found an aging deficit for both the Veh‐Veh and AQ‐Veh groups (*p* < .01 and *p* < .05, respectively), with a trend for an aging deficit for the Veh‐AQ group (*p* = .06), but no significant aging deficit for the AQ‐AQ group (*p* = .8). Thus, these results suggest AQ infusion 1 hr before training and testing mitigates an age‐related context fear memory deficit.

Analysis of grid crossings during the first 60 s of the training session revealed a significant main effect of age [*F*(1, 37) = 20.56, *p* < .001], no effect of infusion [*F*(3, 37) = 1.49, *p* = .23], and no age by infusion interaction [*F*(3, 37) = 2.51, *p* = .07] (Table 2). Pairwise comparisons within each infusion group further revealed that aged Veh‐Veh rats and aged AQ‐Veh rats displayed reduced number of grid crossings relative to their adult counterparts (*p* < .001 and *p* < .01, respectively). This difference does not account for any aging‐related impairments of trace or context fear conditioning, since a reduction in the number of grid crossings suggest a tendency toward less movement, which if anything might mask as more not less freezing in the aged rats.

**Table 2:** Grid crossings and latency to onset of freezing for experiment 2

Group	No. of grid crossings	Latency to onset of freezing (s)	
		Cue test	Context test
Veh‐Veh			
Adult	17.6 ± 1.2	20.3 ± 2.3	32.9 ± 7.9
Aged	9.6 ± 0.9***	20.4 ± 2.0	42.1 ± 7.7
Veh‐AQ			
Adult	15.2 ± 1.5	23.5 ± 6.7	75.5 ± 37.3
Aged	14.8 ± 2.8	23.5 ± 4.9	29.5 ± 15.6
AQ‐Veh			
Adult	18.2 ± 2.3	21.5 ± 3.6	54.5 ± 17.3
Aged	10.0 ± 1.6**	23.5 ± 3.0	60.7 ± 42.8
AQ‐AQ			
Adult	12.8 ± 1.8	20.1 ± 2.7	39.7 ± 8.6
Aged	9.3 ± 1.5	17.3 ± 0.2	47.3 ± 12.2

Note

Values are means ± *SEM*. The number of grid crossings during the first minute of the training session was used to determine the effect of age and infusion on gross baseline activity levels. There was a significant main effect of age, and pairwise comparisons revealed that aged rats from both Veh‐Veh and Veh‐AQ groups made fewer crossings relative to their adult counterparts. Although this suggests these rats may have a tendency to freezing more, this fails to account for the age‐related deficits that were observed during testing. Latency to onset of freezing during the cue and context tests was used to determine whether there were any age‐ or infusion‐related effects on time at which rats began to freeze. Overall, there were no effects of age or infusion on freezing onset during either test.

** *p* < .01

*** *p* < .001

Analysis of latency to onset of freezing during the cue test revealed no effect of age [*F*(1, 37) = 0.006, *p* = .941], no effect of infusion [*F*(3, 37) = 0.63, *p* = .6], and no age by infusion interaction [*F*(3, 37) = 0.13, *p* = .94]. Similarly, analysis of latency to onset of freezing during the context test revealed no effect of age [*F*(1, 37) = 0.18, *p* = .68], no effect of infusion [*F*(3, 37) = 0.51, *p* = .68], and no age by infusion interaction [*F*(3, 37) = 0.91, *p* = .45] (Table 2). This suggests that any age‐ or infusion‐related effects we observed were not due to differences in the amount of time it took for an animal to begin freezing during the test session.

## 4 DISCUSSION

Here, we report that aged F344 male rats are significantly impaired in trace and contextual fear conditioning. Although a single AQ infusion into the dorsal hippocampus 24 hr before conditioning failed to rescue any aging‐related memory deficits, when AQ was infused into the hippocampus 1 hr before both the training and testing sessions the adult and aged rats exhibited comparable levels of freezing to the training context. Thus, intrahippocampal infusions of AQ were able to reverse the context fear memory deficits observed in aged rats.

### 4.1 AQ infusion differentially modulates fear memory deficits in aged rats

Apoaequorin infusion did not alter trace interval freezing in either experiment for adult or aged rats, suggesting trace fear memory is unaffected by AQ infusion. Our infusions were targeted to the dorsal hippocampus, which is known to support trace fear memory (Burman, Starr, & Gewirtz, 2006; Chowdhury, Quinn, & Fanselow, 2005; Esclassan, Coutureau, Di Scala, & Marchand, 2009; Fendt, Fanselow, & Koch, 2005; Guimarais, Gregorio, Cruz, Guyon, & Moita, 2011; Huang, Chiang, Liang, Thompson, & Liu, 2010; McEchron, Bouwmeester, Tseng, Weiss, & Disterhoft, 1998; McEchron, Tseng, & Disterhoft, 2000; Misane et al., 2005; Peters et al., 2009; Pierson, Pullins, & Quinn, 2015; Quinn, Loya, Ma, & Fanselow, 2005; Quinn, Oommen, Morrison, & Fanselow, 2002; Raybuck & Lattal, 2011; Rogers, Hunsaker, & Kesner, 2006; Runyan & Dash, 2005; Seo, Pang, Shin, Kim, & Choi, 2008; Trivedi & Coover, 2006; Wang et al., 2013; Wanisch, Tang, Mederer, & Wotjak, 2005). However, other evidence suggests the circuitry involved in trace fear learning includes several cortical regions in addition to the hippocampus. Impaired trace fear memory is evident following disruption of entorhinal cortex‐hippocampal synaptic transmission (Suh, Rivest, Nakashiba, Tominaga, & Tonegawa, 2011), and following disrupted NMDAR function or enhanced GABA_A_ neurotransmission in the medial prefrontal cortex (Gilmartin & Helmstetter, 2010). Additional evidence suggests a role for perirhinal cortex, as trace fear memory is impaired following NMDA lesions of this brain region (Kholodar‐Smith, Boguszewski, & Brown, 2008). Other evidence indicates ventral hippocampus supports trace fear memory as well (Beeman, Bauer, Pierson, & Quinn, 2013; Cox, Czerniawski, Ree, & Otto, 2013; Czerniawski, Yoon, & Otto, 2009; Esclassan et al., 2009; Gilmartin, Kwapis, & Helmstetter, 2012; Rogers et al., 2006; Yoon & Otto, 2007). Whether aging‐related trace fear memory deficits would be mitigated following AQ infusion targeted to these or other brain regions known to support acquisition or retrieval of trace fear memory remains to be determined.

We found that when AQ was infused 1 hr before conditioning and testing (i.e., AQ‐AQ rats), the context fear memory deficits normally present in aged rats were abolished. In contrast, all other groups demonstrated an aging‐related context fear memory deficit. Although we only observed a trend toward a significant aging‐related context fear deficit in the Veh‐AQ group (Figure 3b), percent freezing in these aged rats was drastically lower compared with aged rats in the AQ‐AQ group (35% vs. 52%, respectively). Thus, context fear memory was selectively enhanced for aged rats that received AQ prior to both the training and testing sessions (see Figure 3d). Future studies will be needed to determine whether this enhancement is long‐lasting (e.g., by assessing behavioral performance during a remote memory test without AQ infusion).

There is a strong link between aging and impaired memory for the training context following trace fear conditioning (Kaczorowski & Disterhoft, 2009; Moyer & Brown, 2006), a memory that is dependent upon dorsal hippocampal function (Chowdhury et al., 2005; McEchron et al., 1998; Misane et al., 2005). Given the evidence suggesting that hippocampal calcium‐binding protein expression is reduced with aging (Dutar, Potier, Lamour, Emson, & Senut, 1991; de Jong et al., 1996; Villa, Podini, Panzeri, Racchetti, & Meldolesi, 1994), there is the possibility that age‐related calcium‐binding protein reduction could contribute to insufficient Ca^2+^ sequestration in this region of the brain. This would weaken hippocampus‐dependent fear memory via impaired physiological or molecular processes.

Synaptic activity‐induced increases of intracellular Ca^2+^ are vital for activation of transcription factors (e.g., CREB) that are critical for memory formation (Silva, Kogan, Frankland, & Kida, 1998). CREB activity appears to be regulated by increased Ca^2+^ levels near the cell membrane, implicating a role for Ca^2+^ sensors like calmodulin and its associated kinases in promoting nuclear CREB activation (Deisseroth, Bito, & Tsien, 1996). Indeed, knockout of the EF‐hand neuronal Ca^2+^ sensor‐1 (NCS‐1) in *C. elegans* suppresses isothermal tracking, a form of experience‐dependent learning, while NCS‐1 overexpression enhances learning (Gomez et al., 2001). In aged animals therefore, the benefit of added Ca^2+^ sensors may be two‐fold: (a) these proteins contribute to sequestration of excess Ca^2+^ that may otherwise trigger cellular toxicity and dysfunction (e.g., Sattler & Tymianski, 2000), and (b) they may play a role in the activation of downstream signaling mechanisms and transcription factors like CREB that ultimately facilitate memory formation. Future work is needed to determine whether either possibility occurs following intrahippocampal AQ infusion. Regardless, our finding that AQ infusion before training and testing alleviates context fear memory deficits in aged rats suggests that this effect may be due to sufficient Ca^2+^ sequestration by the AQ protein.

It is also possible that AQ infusion state‐dependently enhances context fear memory in aged rats, because AQ was infused both before training and testing. State‐dependent learning is often exemplified by enhanced memory retrieval when the brain states during behavioral training and testing are similar (Overton, 1974; Poling & Cross, 1993). There is some evidence to suggest that trace and context fear memory can be modified in a state‐dependent manner rather than through a change in mnemonic processes (Hunt & Barnet, 2016; Jovasevic et al., 2015; Reich, Mohammadi, & Alger, 2008). In our experiments, brain state was affected by infusion. Thus, for there to be evidence of traditional state‐dependent learning, we would expect Veh‐Veh and AQ‐AQ groups to display similar patterns of behavior during the test. That is, since brain state during training and testing is the same for both groups, we would expect enhanced fear retrieval for aged Veh‐Veh and aged AQ‐AQ rats. However, our data demonstrate that a context fear memory deficit is reversed in the aged rats that received AQ before both training and testing, but not for the aged rats that received vehicle before both sessions. Our findings instead reflect a state‐dependent facilitation of context fear memory that requires AQ, and not vehicle, to be on board during both the training and the testing sessions.

### 4.2 Calcium dysregulation and the AQ protein

Our data are consistent with other studies that show Ca^2+^ regulation is beneficial for cognitive function in aged animals. Most of these studies utilize substances that impede Ca^2+^ entry into cells, either from the extracellular space via L‐VDCCs (Deyo et al., 1989; Veng et al., 2003), or from intracellular stores via ryanodine receptors (Gant et al., 2015; Hopp et al., 2014). Additional evidence suggests regulation of calcium‐dependent physiological mechanisms in the hippocampus can mitigate age‐related neuronal dysfunction. Specifically, reduced expression of small‐conductance calcium‐activated potassium channel (SK3) expression using SK3 antisense oligonucleotides enhances LTP and improves trace fear memory in aged mice (Blank, Nijholt, Kye, Radulovic, & Spiess, 2003). Although these studies suggest that preventing Ca^2+^ entry into the cytosol improves cognitive and physiological function during aging, it remains unclear if sequestration of free Ca^2+^ ions already located in the intracellular space would also effectively restore cognitive function in aged animals. While synaptic plasticity in aged hippocampal CA1 neurons is enhanced following application of the Ca^2+^ chelators BAPTA‐AM and EGTA‐AM (Ouanounou, Zhang, Charlton, & Carlen, 1999), the effects of overexpression of these or other calcium‐buffering mechanisms on cognitive function in aged animals have not been fully explored. Our goal was to expand upon this area of research by infusing the calcium‐binding protein AQ directly into the aged hippocampus to determine its potential for ameliorating age‐related cognitive deficits.

Previous research from our laboratory indicates that cell death following an in vitro ischemic insult is reduced when AQ is infused into the dorsal hippocampus either 24 or 48 hr prior to the insult (Detert et al., 2013). Because excitotoxicity and cell death following ischemia can be enhanced by excessive Ca^2+^ influx (Choi, 1985; Simon, Swan, Griffiths, & Meldrum, 1984), application of the AQ protein likely confers neuroprotection via its calcium‐binding capabilities. However, Western blots indicate the AQ protein is present in hippocampal tissue 1 hr after infusion, but it is greatly diminished or absent by 1 or 2 days later (Detert et al., 2013). This suggests the neuroprotection conferred by the AQ protein in these experiments may be due its influence on other signaling molecules involved in neuroprotection (e.g., cytokines and/or chemokines; Detert et al., 2013). Thus, AQ‐induced neuroprotection in an ischemic model may not directly map onto a behavioral model like that used in the current study. Instead, it may be more likely to observe the effects of AQ on overt learning and memory tasks such as trace fear conditioning if behavioral manipulations occur when protein presence is greatest (i.e., 1 hr after infusion), allowing for a more direct targeting of critical aspects of learning, such as acquisition or consolidation. Our observation in experiment 2 that aged and adult rats display similar contextual freezing closely following AQ infusion is consistent with this idea. Overall, these data suggest direct intrahippocampal AQ infusion delivered close in time to behavioral training and testing rescues an aging‐related contextual fear retrieval deficit.

## 5 CONCLUSIONS

Aging‐related cognitive decline is thought to emerge from a variety of alterations that occur during normal aging, including dysregulated Ca^2+^ homeostasis (Khachaturian, 1987, 1994; Landfield, 1987). The present study demonstrates that aged rats are significantly impaired in both trace and contextual fear conditioning, consistent with previous studies (Dulka et al., 2020; Kaczorowski et al., 2012; McEchron et al., 2004; Moyer & Brown, 2006; Villarreal et al., 2004). Interestingly, AQ infusions into the dorsal hippocampus could reverse the aging‐related deficits in contextual fear conditioning, but only when the infusions were given 1 hr prior to both the training and the testing session. No significant improvements in trace fear conditioning were observed in aged rats in either of the experiments. These data suggest that when delivered in close proximity to both training and testing, a dose of AQ that is neuroprotective can also mitigate aging‐related memory deficits.

## CONFLICT OF INTEREST

The authors declare no conflicts of interest.

## AUTHOR CONTRIBUTION

J.M. and V.E. designed the study; V.E. and C.S. performed experiments; V.E. and J.M. wrote the manuscript.

### Peer Review

The peer review history for this article is available at https://publons.com/publon/10.1002/brb3.1832.

## Data Availability

The data that support the findings of this study are available from the corresponding author upon reasonable request.
